# Dynamics of Alloplastic Bone Grafts on an Early Stage of Corticotomy-Facilitated Orthodontic Tooth Movement in Beagle Dogs

**DOI:** 10.1155/2014/417541

**Published:** 2014-09-03

**Authors:** Hyung-Joo Choi, Dong-Yeol Lee, Tae-Woo Kim

**Affiliations:** ^1^Seoul National University School of Dentistry & Dental Research Institute, Seoul 110-768, Republic of Korea; ^2^Seoul Barun Dental Clinic, Anyang, Gyeonggi-do 431-050, Republic of Korea; ^3^Department of Oral and Maxillofacial Pathology and Research Center for Tooth & Periodontal Regeneration (MRC), School of Dentistry, Kyung Hee University, Seoul 130-701, Republic of Korea

## Abstract

Alveolar augmented corticotomy is effective in accelerating orthodontic tooth movement, but the effect only lasts for a relatively short time. Therefore, the purpose of this study was to investigate the underlying biology of the immediate periodontal response to orthodontic tooth movement after a corticotomy with alloplastic bone grafts. The results demonstrated that measurable tooth movement began as early as 3 days after the intervention in beagle dogs. Based on the results and histological findings, augmented corticotomy-facilitated orthodontic tooth movement might enhance the condition of the periodontal tissue and the stability of the outcomes of orthodontic treatment.

## 1. Introduction

New appliances, materials, and mechanics of orthodontic treatment are being developed every day, but much of the biology of orthodontic tooth movement still needs to be clarified. Many adjunctive modalities are available to accelerate orthodontic tooth movement in humans, such as corticotomy [1–10], distraction osteogenesis [11], mechanical vibration [12], medication with local prostaglandins [13], and low-level laser treatment [14, 15]. Among these interventions, corticotomy is known to be the most effective means to accelerate orthodontic tooth movement [7, 16]. In experiments that used a split-mouth design, with corticotomy performed on one side and the other side serving as the control, the velocity of the tooth movement was accelerated on the corticotomy side [1, 17–19], and the amount of movement doubled over the duration of the experiments [17–20].

The initial microscopic changes and early application of orthodontic force have been emphasized in corticotomy-facilitated orthodontic tooth movement [16], and it has been hypothesized that a corticotomy or an osteotomy can lead to intensified osteoclastic activity resulting in local osteopenia and increased bone remodeling [2, 17, 21, 22]. To date, however, the immediate periodontal response has not been fully elucidated. Most researchers have either studied the small animals such as rats, cats, or rabbits [2, 6, 10, 20–22], for relatively long periods of 6–12 weeks [4, 5], or carried out gross observational studies with no histologic measurements [1, 2, 9, 19, 23].

The purpose of this study was to investigate the immediate periodontal response to a corticotomy with alloplastic bone grafts in beagle dogs.

## 2. Materials and Methods

### 2.1. Animal Subjects

Five adult male beagle dogs, weighing 10–13 kg, were used in the experiment, and their selection, care, and preparation, together with the surgical protocol, were carried out according to the guidelines for animal experiments (IRB no. KHMC-IACUC2012-024). They were caged separately under regulated conditions and fed a normal diet and water* ad libitum* to secure the experimental orthodontic appliances.

For the preparation processes and surgical procedures, the animals were anesthetized with a mixture of tiletamine-zolazepam and xylazine, via intramuscular and intravenous injections using a catheter in the vessel of the ear.

### 2.2. Study Preparation

**Figure 1:**
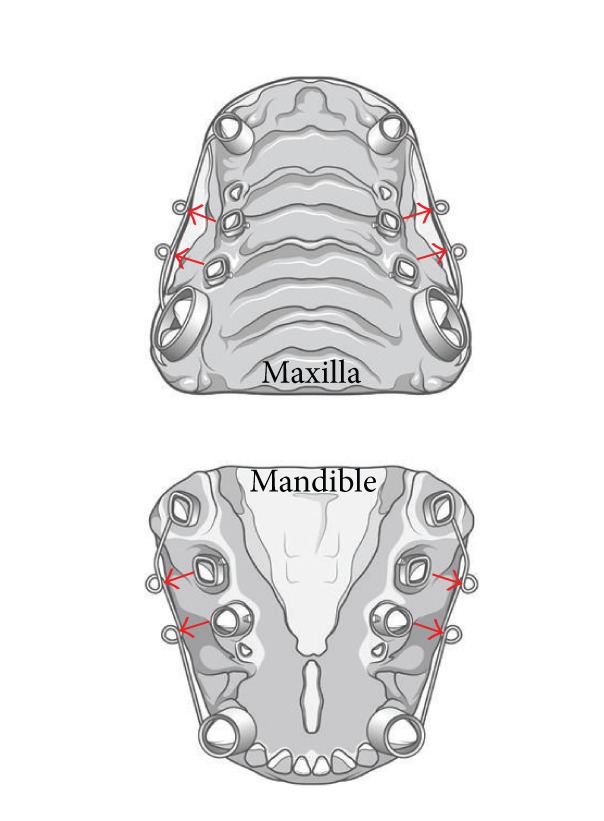
Orthodontic appliances were custom-made for each study model. The canine and fourth premolar teeth were banded to form an anchor tooth, and a *Ø* 0.9 mm stainless steel wire was welded on the buccal surface of the bands. The second and third premolars were banded with a lingual button. After 4 weeks, the animals were anesthetized to fit the orthodontic appliances to the teeth. The arrows indicate the direction of force.

Alginate impressions of each beagle were taken to make study models, and orthodontic appliances were custom-made for each model. The canine and fourth premolar teeth were banded to form anchor teeth, and a *Ø* 0.9 mm stainless steel wire was welded onto the buccal surface of the bands. The second and third premolars were banded with a lingual button. After 2 weeks, the animals were anesthetized to fit the orthodontic appliances to the teeth (Figure 1).

### 2.3. Surgical Procedures for the Corticotomy and Alloplastic Bone Graft

**Figure 2:**
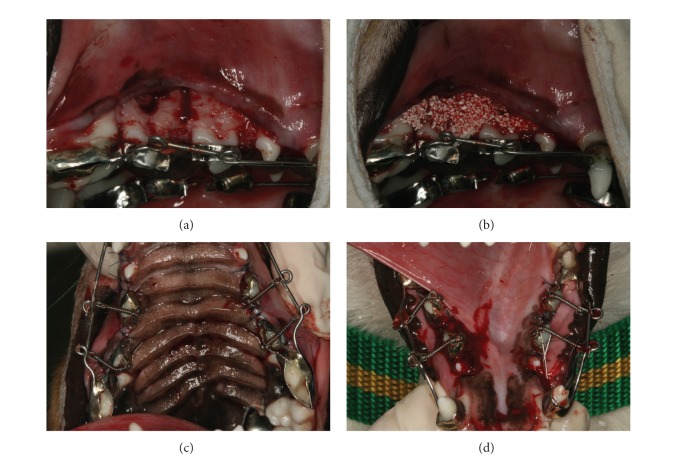
Surgical procedure. (a) Corticotomy: the circumscribing corticotomy was performed with a round bur (*Ø* 1.5 mm) under sterile saline irrigation. (b) MBCP^+^ (Biomatlante, Vigneux de Bretagne, France), composed of 20% hydroxyl apatite and 80% *β*-tri-calcium phosphate, was used as the alloplastic bone graft material. The graft bone was applied soaked in blood; 1 g of the MBCP^+^ was grafted on the surgical surface. (c) A closed coil spring made of nickel-titanium shaped memory wire of 200 g force was applied to the second and third premolars in a buccolingual direction in the maxilla and (d) in the mandible.

Under general anesthesia, 2% lidocaine with 1 : 100,000 epinephrine was also infiltrated to the surgical sites. An intrasulcus incision was performed with a no. 12 blade from the canine tooth to the first molar, and a full-thickness flap was lifted. The circumscribing corticotomy (Figure 2(a)) was performed with a round bur (*Ø* 1.5 mm) under sterile saline irrigation. Alloplastic bone material (MBCP+, Biomatlante, Vigneux de Bretagne, France), composed of 20% hydroxyl apatite and 80% *β*-tri-calcium phosphate, was used for the graft. The graft bone was soaked with blood, and 1 g of the MBCP+ was grafted onto the surgical surface (Figure 2(b)). The mucoperiosteal flaps were repositioned and sutured with 5–0 nylon and primary closure was obtained (Figure 2(c)). A closed coil spring made of nickel-titanium shape memory wire of 200 g force was applied to the second and third premolars in a buccolingual direction (Figure 2(d)).

**Figure 3:**
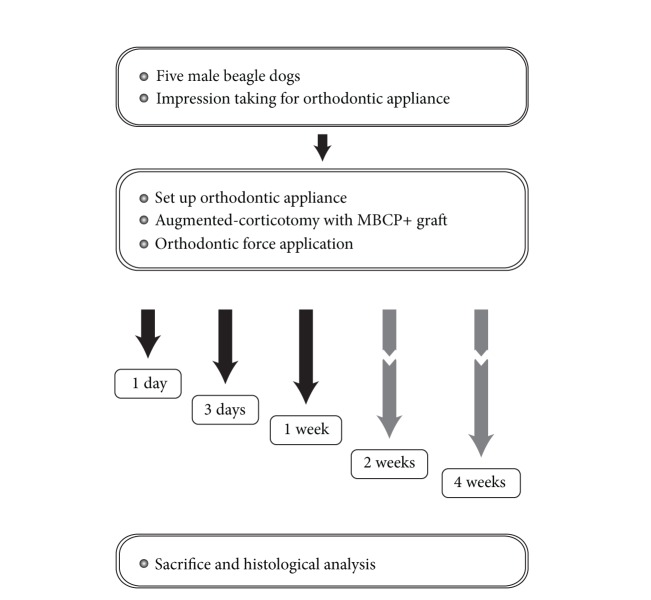
Schematic diagram describing the experiment design.

All of the surgical procedures were performed under sterile conditions to prevent infection. After surgery, antibiotics and anti-inflammatory analgesics were administered by intramuscular injection twice a day for 6 days. A 1% chlorhexidine-gluconate solution dressing was applied simultaneously for infection control. A soft diet was supplied for 1 or 2 weeks and then a normal diet. Mechanical plaque control was performed once a week. The animals were euthanized with an overdose of thiopental sodium after 1 day, 3 days, 1 week, 2 weeks, and 4 weeks following the surgery (Figure 3).

### 2.4. Histological Processing and Analysis

Following a predetermined time schedule, after the animals were killed, their maxillae and mandibles were dissected, and the sections containing the canine to the fourth premolar teeth were retrieved. The block specimens were rinsed in sterile saline and immediately immersed in 10% neutral-buffered formalin fixatives for 14 days. The block specimens were large, and rapid decalcification was performed for 6 days using 5% nitric acid because it is sufficiently strong [24]. Had this study been designed for immunohistology, the use of ethylene-diamine-tetra-acetic acid or 10% aqueous or formic acid would have been suitable [25], but this was not the case [5, 26]. The specimens were then dehydrated through a series of ethanol solutions of increasing concentrations and embedded in paraffin. Buccolingual sections were sliced with a microtome set at 5 *μ*m and stained with the Masson's trichrome solution. One slide was processed per experimental tooth.

Histological examinations were conducted using a light microscope (Olympus BX 51, Olympus, Tokyo, Japan) equipped with a DP controller 3.2.276.2 and DP manager 3.1.1.208 (Olympus, Tokyo, Japan). After microscopic examination, a photograph of each slide was taken with a digital camera (Olympus DP 71, Olympus, Tokyo, Japan). With imaging software (cellSens version 1.6, Olympus), we measured the buccal tipping angle (°) and distance (*μ*m). The buccal tipping angle was measured from the reversal line of the lingual/palatal bone wall to the lingual/palatal root surface. The buccal tipping distance was measured from the lingual/palatal alveolar crest to the shortest lingual/palatal root surface. A total of 40 slides were fabricated and examined. After taking photographs, the amount of tooth movement was measured three times in each slide for angular changes and linear displacement.

### 2.5. Statistical Data Analysis

Statistical data analysis was performed using the R programming language [27]. The data on tooth movement did not fulfill the parametric conditions of normality and equality of variance after the D'Agostino normality test was performed. We therefore conducted the Kruskal-Wallis rank sum test to determine whether there existed a significant between-group difference in general and the Wilcoxon test to find a significant pair between two groups. The Bonferroni correction and the Type I error were applied to counteract the problem of multiple comparisons. The data were analyzed with a confidence level of 95%.

## 3. Results

### 3.1. Clinical Findings

**Figure 4:**
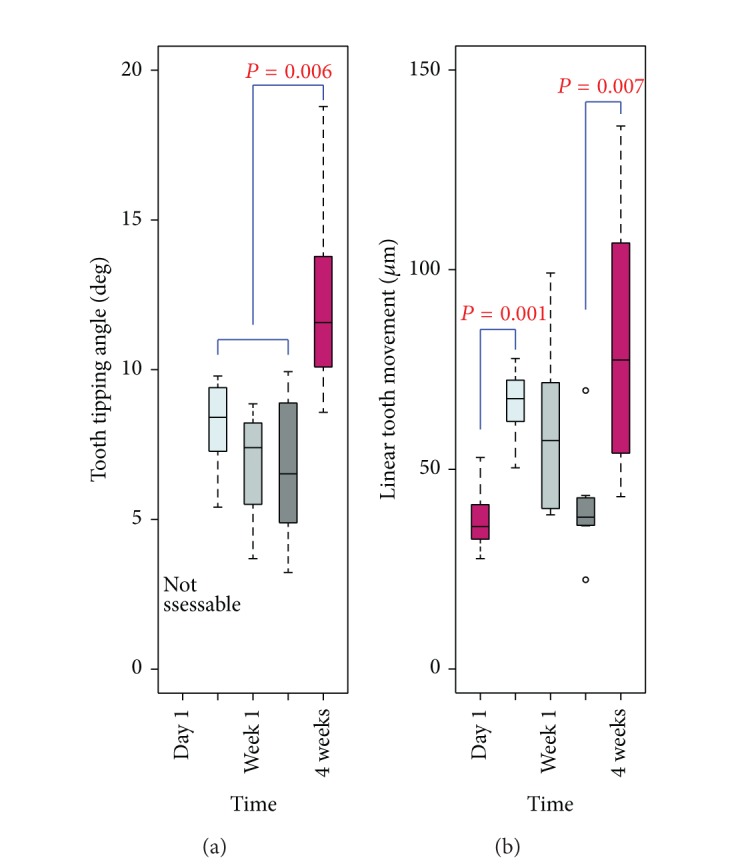
Box plots for the tooth movement measurements. (a) No statistically significant difference in the angular tooth movement was observed over time. (b) However, the linear measurements demonstrated statistically significant differences between 1 day and 3 days and between 2 weeks and 4 weeks after the start of the experiment.

Figure 4 shows the amount of tooth movement for each beagle dog. Due to the minimal tooth movement, the buccal tipping angle at 1 day could not be measured. No significant difference was observed between the teeth in the maxilla and those in the mandible or in the right or left part of the dentition. When the tipping movement was measured from the angular changes, no statistically significant difference was found among the experimental groups (Figure 4(a)). However, the linear measurements demonstrated statistically significant differences between 1 day and 3 days and between 2 weeks and 4 weeks after the start of the experiment (Figure 4(b)).

### 3.2. Histological Observations

**Figure 5:**
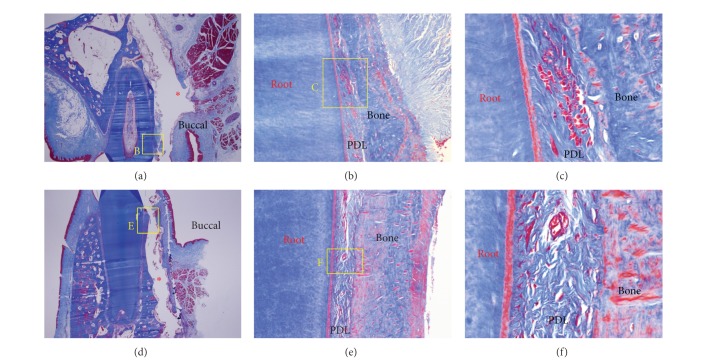
Microphotograph of a buccopalatal/lingual section of the 1-day experiment. (a) Maxilla. (b) Higher magnification of (a). On the pressure side, the PDL was compressed. (c) Higher magnification of (b). Extravasation of RBC was observed. (d) Mandible. (e) Higher magnification of (d). Compression of the PDL was shown, and reduced capillaries were identified. (f) Higher magnification of (e). The number of cells was reduced, and grafted MBCP+ particles were lost in the process of making the histological section; the red ∗ in (a) and (d) indicates empty spaces that were occupied by MBCP+ graft particles. Masson's trichrome stain. Original magnification was ×12.5 for (a) and (d), ×100 for (b) and (e), and ×400 for (c) and (f).

After 1 day of orthodontic movement, a microphotograph of the buccopalatal/lingual section (Figure 5) showed compression of the periodontal ligament (PDL) (Figure 5(b)), extravasations of red blood cells (RBC) (Figure 5(c)), and reduced capillaries (Figure 5(e)) on the pressure side. No significant finding was observed on the tension side.

**Figure 6:**
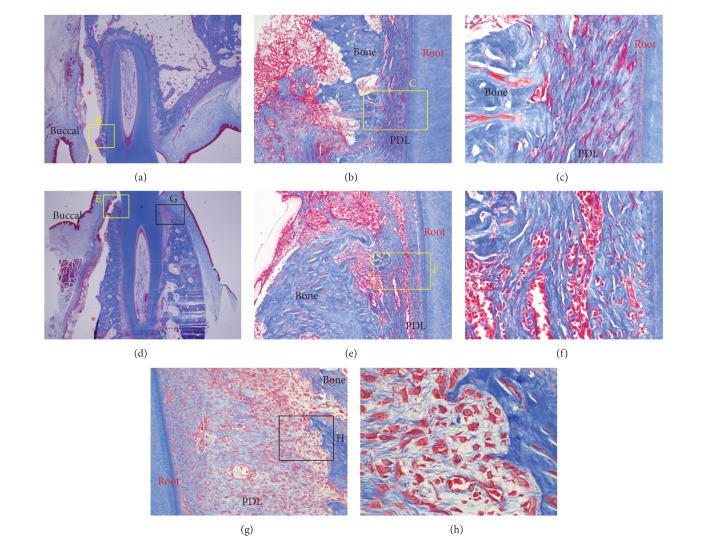
Microphotograph of a buccopalatal/lingual section of the 3-day experiment. (a) Maxilla. (b) Higher magnification of (a). On the pressure side, the PDL was more severely compressed. (c) Higher magnification of (b). Few cells were observed in the PDL space. (d) Mandible. (e) Higher magnification of (d). In common with the maxilla, the PDL was severely more compressed on the pressure side. (f) Higher magnification of (d). Extravasation of RBC was observed in the PDL space. (g) Tension side at the lingual bone crest. The tension side showed abundant cells compared with the pressure side. (h) Higher magnification of (g). Active osteoblasts forming new bone were observed. Grafted MBCP+ particles were lost in the process of making the histological section; the red ∗ in (a) and (d) indicates empty spaces which were occupied by MBCP+ graft particles. Masson's trichrome stain. Original magnification was ×12.5 for (a) and (d), ×100 for (b), (e), and (g), and ×400 for (c), (f), and (h).

At 3 days, the PDL was more severely compressed and fewer cells were found in the PDL space (Figure 6(c)) on the buccal pressure side. The tension side at the lingual alveolar bone crest contained more cells than the pressure side and active osteoblasts forming a new bone (Figure 6(h)).

**Figure 7:**
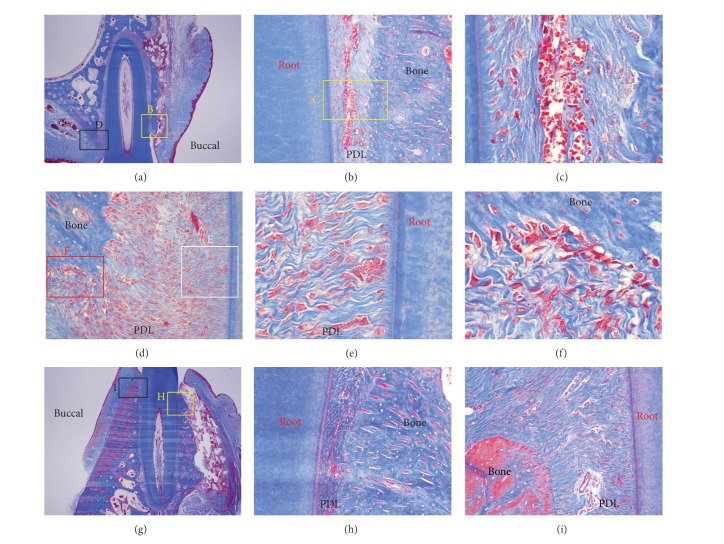
Microphotograph of a buccopalatal/lingual section of the 1-week experiment. Most of the grafted MBCP+ particles were well maintained. (a) Maxilla. (b) Higher magnification of (a). On the pressure side, the PDL space was wider than at 3 days. (c) Higher magnification of (b). Extravasation of RBC was observed in the PDL space. (d) Tension side at the palatal bone crest. The tension side showed abundant cells compared with the pressure side and a widened PDL space. (e) Higher magnification of (d). Abundant PDL fibroblasts were seen. (f) Higher magnification of (d). Active osteoblasts forming a new bone were observed. (g) Mandible. Most of the grafted MBCP+ particles were well maintained. (h) Higher magnification of (g). Pressure side. The PDL was compressed. (i) Higher magnification of (g). The tension side showed a widened PDL space. Masson's trichrome stain. Original magnification was ×12.5 for (a) and (g), ×100 for (b), (d), (h), and (i), and ×400 for (c) and (f).

At 1 week after the start of the experiment, most of grafted MBCP+ particles were well maintained (Figure 7). On the pressure side, the PDL space was slightly widened compared with that at 3 days (Figure 7(b)), and the tension side contained abundant PDL fibroblasts and active osteoblasts (Figures 7(d), 7(e), and 7(f)).

**Figure 8:**
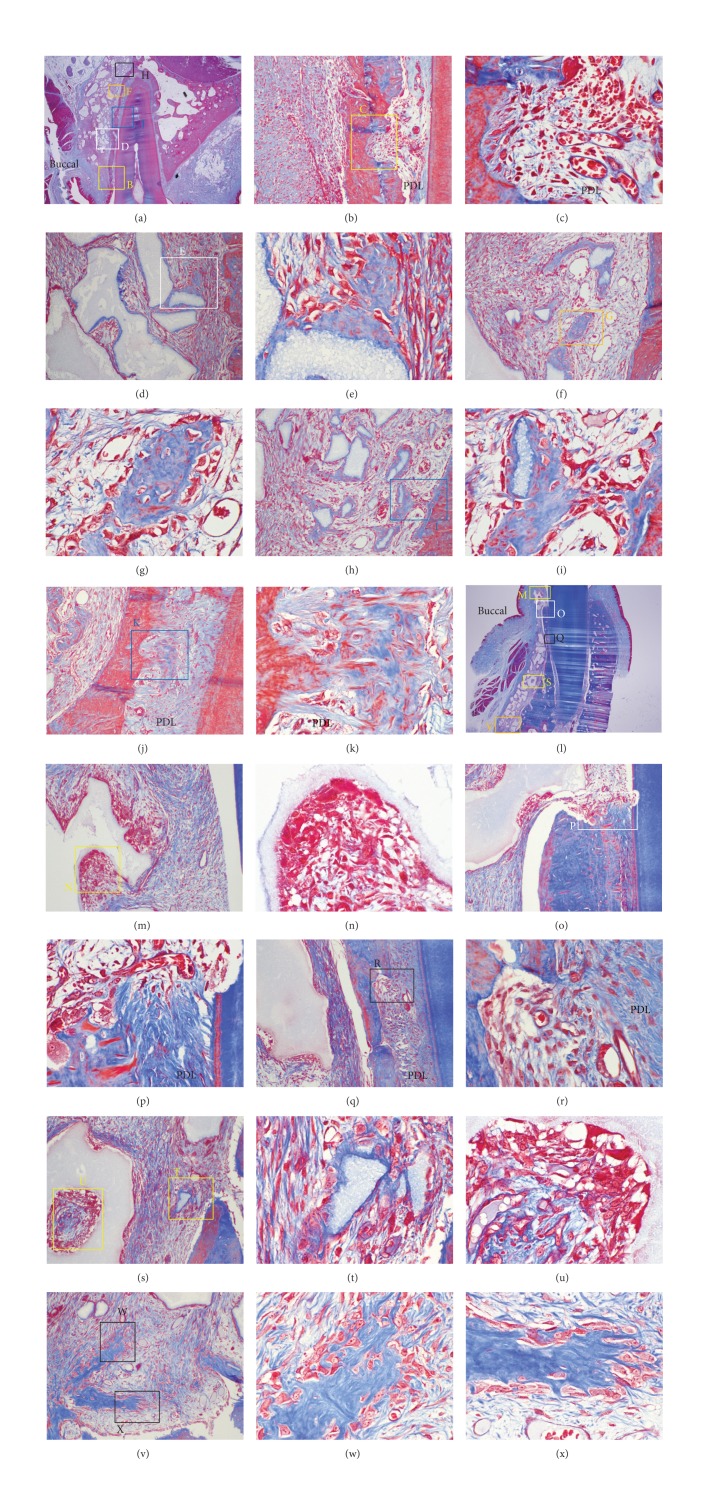
Microphotograph of a buccopalatal/lingual section of the 2-week experiment. (a) Maxilla. Most of the grafted MBCP+ particles were well maintained. (b) Higher magnification of (a) at the crest area. Undermining resorption on the pressure side was observed. (c) Higher magnification of (b). Extravasation of RBC was observed in the PDL space. Resorption bays, which indicate undermining resorption on the pressure side, were also observed. (d) Higher magnification of (a) at the buccal bone surface. New bone formation surrounding the grafted MBCP+ particles was observed. Grafted MBCP+ particles were bridged by newly formed bone. (e) Higher magnification of (d). Abundant osteoblasts were forming new bone. (f) Higher magnification of (a). Osteoblasts were forming a new bone island. (h) Higher magnification of (a). Grafted particles encircled by new bone were bridged with the buccal bone surface. (j) Higher magnification of (a). New bone was formed in the PDL space at the buccal tension area. New bone was formed from the bone. (l) Mandible. Most of the grafted MBCP+ particles were well maintained. (m) Higher magnification of (l) at the crest area. Grafted particles were resorbed by osteoclasts. (n) Higher magnification of (m). (o) Higher magnification of (l). (p) Higher magnification of (l). Bone and root surface resorption by osteoclasts were observed. (q) Higher magnification of (l). Undermining resorption at the buccal bone in the PDL area was observed. (r) Higher magnification of (q). Many osteoblasts filled the resorption bay. (s) Higher magnification of (l). Active new bone formation was found at the buccal bone surface in the apical area. (t) Higher magnification of (s). Many osteoblasts were forming a new bone encircling the grafted MBCP+ particles. (u) Higher magnification of (s). Osteoclastic and osteoblastic activities were both observed. (v) Higher magnification of (l). New bone islands were formed. (w) Higher magnification of (v). (x) Higher magnification of (v). Abundant osteoblasts were actively forming new bone islands. Masson's trichrome stain. Original magnification was ×12.5 for (a) and (l); ×100 for (b), (d), (f), (h), (j), (m), (o), (q), (s), and (v), and ×400 for (c), (e), (g), (i), (k), (n), (p), (r), (t), (u), (w), and (x).

At 2 weeks, undermining resorption and a resorption bay were observed on the buccal pressure side (Figures 8(b), 8(c), 8(q), and 8(l)). In contrast, on the buccal tension side, new bone formation surrounding and bridging the MBCP+ particles was seen (Figures 8(d), 8(j), and 8(f)) due to abundant osteoblasts (Figures 8(e) and 8(k)).

**Figure 9:**
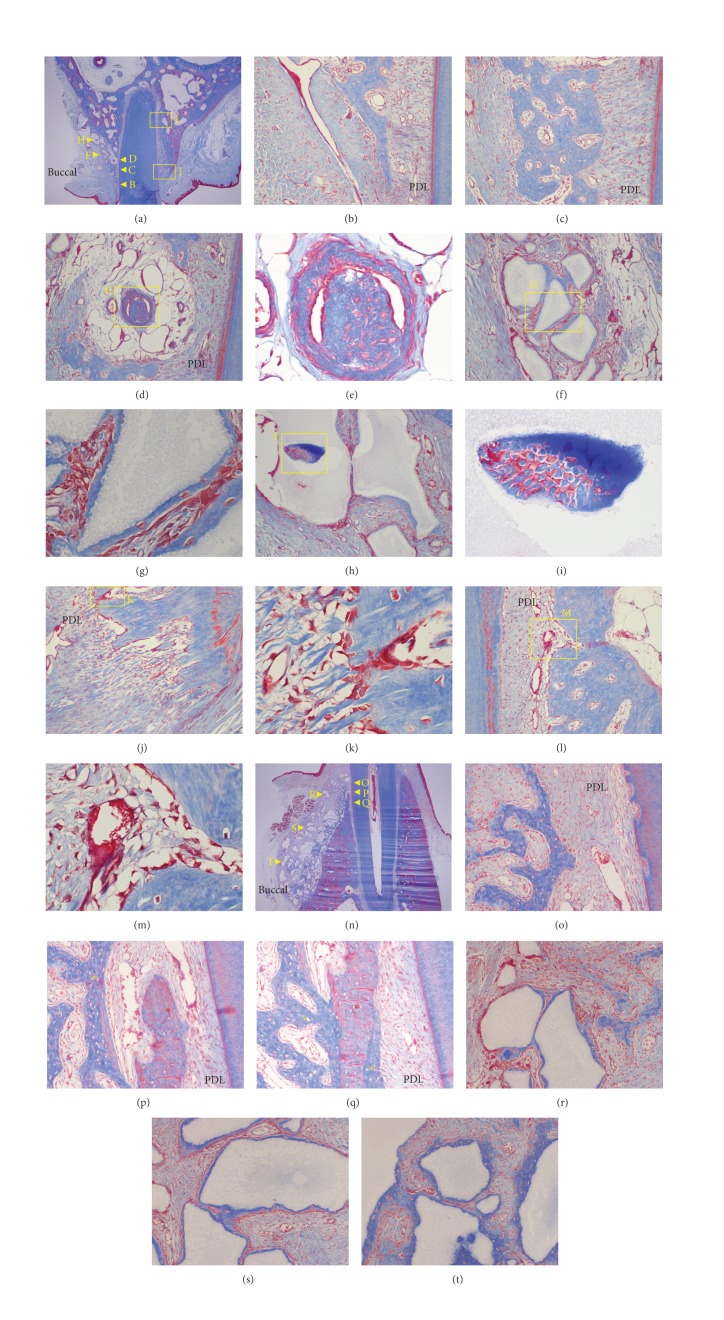
Microphotograph of a buccopalatal/lingual section of the 4-week experiment. (a) Maxilla. Most of the grafted MBCP+ particles were well maintained. New bone formation along the PDL formed a new buccal bone wall. ((b) and (c)) Higher magnification of (a) at the crest area. (d) Higher magnification of (a). The bone-derived mesenchymal matrix bordered the PDL-derived mesenchymal matrix. A new bone island was formed in the center of the bone-derived mesenchymal matrix. (e) Higher magnification of (d). Osteoblasts and osteocytes were observed. (f) Higher magnification of (a). The grafted MBCP+ particles were bridged with newly formed bone in the bone-derived mesenchymal matrix on the buccal side. (g) Higher magnification of (f). Entrapped osteocytes and aggregated osteoblasts were observed. (h) Higher magnification of (a). (i) Higher magnification of (h). New bone was formed in the center of the grafted MBCP+ particles at the buccal side. ((j) and (l)) Higher magnification of (a). The palatal tension side at the crestal (j) and apical (l) areas. ((k) and (m)) Higher magnification of (j) and (l), respectively. Aggregated osteoblasts and active form of osteoblasts were seen. (n) Mandible. (o) Higher magnification of (n) at the crestal area. New bone forming a buccal bone wall and crest was observed. (p) Higher magnification of (n). Native bone (red star) and new bone (yellow star). (q) Higher magnification of (n). New bone (yellow star) was formed on the outer and inner surface of the native bone (red star). ((r), (s), and (t)) Higher magnification of (n). The outer portion of the bone-derived mesenchymal matrix. Grafted MBCP+ particles were bridged by the newly formed bone. Masson's trichrome stain. Original magnification was ×12.5 for (a) and (n), ×100 for (b), (c), (d), (f), (h), (j), (l), (o), (p), (q), (r), (s), and (t), and ×400 for (e), (g), (i), (k), and (m).

At 4 weeks after the start of the experiment, new bone formation along the PDL formed a new buccal bone wall on the pressure side (Figure 9(a)). Also, a new bone island was formed in the center of the bone-derived mesenchymal matrix (Figure 9(d)), and osteoblasts and osteocytes were observed (Figure 9(e)). On the buccal side, grafted MBCP+ particles were bridged with newly formed bone in the bone-derived mesenchymal matrix. Entrapped osteocytes and aggregated osteoblasts were observed (Figure 9(g)). The palatal crestal (Figure 9(j)) and apical (Figure 9(l)) tension sides showed aggregated osteoblasts and active forms of osteoblasts, and a new bone-forming buccal bone wall and crest were observed (Figure 9(o): native bone (red star) and new bone (yellow star)). New bone was formed on the outer and inner surfaces of the native bone (Figures 9(n) and 9(q)), and the outer portion of the bone-derived mesenchymal matrix could be seen (Figure 9(t)).

## 4. Discussion 

The biological mechanism by which the tooth movement is facilitated after a corticotomy has been suggested to be mediated by a regional acceleratory phenomenon [28, 29], which might boost the appearance of the macrophages that eliminate the hyaline as early as 1 week after the application of orthodontic force [17, 21]. For this reason, we designed an experiment to determine immediate periodontal responses, which represents the first study to observe the immediate effect of corticotomy-facilitated orthodontic tooth movement. Two studies previously reported observations made 3 days after a corticotomy in rats [2, 22]. However, the results obtained in small animals may differ from those in larger animals [16]. We believe that the current study is unique because we observed the histological responses of periodontal tissue as early as 1 day, 3 days, 1 week, 2 weeks, and 4 weeks after a corticotomy and force application for orthodontic tooth movement in larger animals.

In most experiments in dogs or rats, corticotomy-facilitated tooth movement was observed at a rate of about 1 mm per month, which was almost double that observed on the control side [17–20]. In this study, we observed significant tooth movement within 3 days (Figure 4). We measured the width of the PDL at the crest of the lingual/palatal sides, which does not represent the direct distance of clinical tooth movement. The significant tooth movement observed 4 weeks after the start of the experiment was to be expected, but this obviously occurred earlier in the experiment. The tooth movement measurements demonstrated a similar pattern in angular changes and linear displacement, as shown in Figure 4. We conjectured that this might imply that the pattern of rapid tooth movement was not a bodily translation in general but mostly occurred through tipping of the tooth. Therefore, during clinical orthodontic treatment, methods to control for unwanted tipping should also be considered.

Despite the similar pattern between the angular and linear changes, the linear measurements exhibited a statistically significant difference while the angular changes did not. Linear measurements are assessed between two points while degrees of angles are measured between three points, from which a variation in angular measurements can be produced. This may be the cause of the larger variation in angular measurements than in linear measurements.

A typical cell-free zone on the pressure side and an inflammatory reaction were seen on the 1- and 3-day slides (Figures 5 and 6). A force of 200 g may be strong for tipping movement, but it was difficult to determine the hyalinization layer in the 1- and 3-day slides. The corticotomy was probably responsible for this, but the underlying mechanism was not clear. The progenitor cells regarded as osteoblasts in the 3-day slides showed strong cellular activity.

On the 1-week slide, we observed that the PDL space was widened at the pressure side compared with that of the 3-day slide (Figure 7(b)). This could be explained by undermining resorptions. On the tension side, active osteoblasts forming a new bone were observed. This new bone formation is a common phenomenon in orthodontic tooth movement.

Interestingly, new bone surrounding the graft materials was observed on the compression side on the 2-week slides (Figure 8(d)) and at the buccal sides distant from PDL (Figures 8(s), 8(t), and 8(u)). Graft particles were bridged by newly formed bone and osteoclastic and osteoblastic activities were both seen (Figure 8(u)).

From the findings of the 4-week sections, the new bone formation in the center of the MBCP+ graft material on the buccal side was very distinctive (Figures 9(h) and 9(i)), and many entrapped osteocytes and aggregated osteoblasts were observed (Figure 9(g)). However, the cause of this new bone formation on the buccal sides around the graft materials was not certain. Further studies could provide the answer.

The slides taken at 2 and 4 weeks did not show a distinctive loss of periodontal attachment, and the small areas of root resorption that were seen were not significant.

Orthodontic patients have complained about the length of their treatment, and it has become necessary to develop adjunctive methods to tackle this problem [1, 3, 7–15]. Alveolar corticotomy is effective in accelerating orthodontic tooth movement [2, 7, 16]. However, according to pertinent studies [1, 16, 19], the regional acceleratory phenomenon only persists for about 4 months, after which the tooth movement rate returns to normal. To make use of this “window” period in an effective, efficient, and efficacious way, it is imperative to understand the underlying biology of early periodontal responses to tooth movement after augmented corticotomy and to develop appropriate clinical procedures. Also, from the clinical point of view, it is important to determine how to reduce the length of total treatment time. When should the corticotomy be performed? How often should orthodontic force be applied? What magnitude of force would be optimal? These are still widely open questions that are worthy of further investigation.

Although there is no doubt that this procedure could align teeth within a shorter period of time [1, 3, 8, 16, 30–33], at the present time, we cannot expect augmented corticotomy-facilitated orthodontic tooth movement to reduce the entire length of orthodontic treatment noticeably in adult patients. In addition, there is a paucity of information in the available research to assert that grafting enhances the stability of orthodontic treatment [16]. To prove this, well-designed randomized controlled clinical trials should be performed.

The combination of corticotomy with an alveolar graft was introduced by Wilcko et al. and is referred to as accelerated osteogenic orthodontics or periodontally accelerated osteogenic orthodontics [8, 9, 34]. They asserted that bone grafting of the labial and lingual cortical bones would increase the stability of orthodontic treatment, enhance the range of possible tooth movements, increase alveolar bone volume, and provide a more structurally stable periodontium. However, no convincing scientific or histological evidence was available other than clinical reports. In some case reports, increased volume of bone around the alveolus was observed after bone grafting [3, 5, 9]. The present study supported the hypothesis that tooth movement with an augmented corticotomy might enhance orthodontic tooth movement, because we found new bone formation at the buccal surface. However, it focused on initial responses, and further investigation is needed to clarify its findings.

Tooth movement that was thought to be difficult or impossible to produce in the past has become possible due to the development of orthodontic mechanics and new appliances and materials. Therefore, orthodontic treatment is mainly limited by the scope of the alveolar bone, and orthognathic surgery is required when this limit is exceeded. If an augmented corticotomy could increase the alveolar bone volume, this would help patients who have a limited amount of supporting alveolar bone.

Augmented corticotomy surgery is not free from some morbidity. It also requires a skilled clinician, and there may be some discomfort to patients and additional costs.

## 5. Conclusions

The findings from this study suggest that measurable tooth movement starts as early as 3 days after augmented corticotomy-facilitated orthodontic treatment and that this procedure might enhance the condition of periodontal tissue and the stability of orthodontic treatment outcomes.
